# Dynamic, mating-induced gene expression changes in female head and brain tissues of *Drosophila melanogaster*

**DOI:** 10.1186/1471-2164-11-541

**Published:** 2010-10-06

**Authors:** Justin E Dalton, Tanvi S Kacheria, Simon RV Knott, Matthew S Lebo, Allison Nishitani, Laura E Sanders, Emma J Stirling, Ari Winbush, Michelle N Arbeitman

**Affiliations:** 1 Section of Molecular and Computational Biology, Department of Biological Sciences, University of Southern California, Los Angeles, California 90089, USA

## Abstract

**Background:**

*Drosophila melanogaster *females show changes in behavior and physiology after mating that are thought to maximize the number of progeny resulting from the most recent copulation. Sperm and seminal fluid proteins induce post-mating changes in females, however, very little is known about the resulting gene expression changes in female head and central nervous system tissues that contribute to the post-mating response.

**Results:**

We determined the temporal gene expression changes in female head tissues 0-2, 24, 48 and 72 hours after mating. Females from each time point had a unique post-mating gene expression response, with 72 hours post-mating having the largest number of genes with significant changes in expression. At most time points, genes expressed in the head fat body that encode products involved in metabolism showed a marked change in expression. Additional analysis of gene expression changes in dissected brain tissues 24 hours post-mating revealed changes in transcript abundance of many genes, notably, the reduced transcript abundance of genes that encode ion channels.

**Conclusions:**

Substantial changes occur in the regulation of many genes in female head tissues after mating, which might underlie aspects of the female post-mating response. These results provide new insights into the physiological and metabolic changes that accompany changes in female behaviors.

## Background

Successful reproduction in *Drosophila melanogaster *requires the interplay of behavioral repertoires performed by males and females, which include male courtship, female receptivity, copulation, and female post-mating responses. Females are biochemically poised to respond to sperm, proteins, and other molecules transferred during copulation, which induce the post-mating response. This response, which lasts about a week, includes reduced receptivity to mating, metabolic changes, increased immunity and the physiological changes that accompany sperm storage, fertilization and egg-laying [reviewed in [1,2]].

Accessory gland proteins (Acps), which are synthesized in the male accessory gland and transferred to females in the seminal fluid during copulation, act together to induce the post-mating response (reviewed in [1-3]). Of the Acps identified, sex peptide (SP) appears to be one of the primary triggers of the post-mating response. Injection of biochemically purified SP or ectopic expression of the gene that encodes SP in virgin females induces increased egg laying and reduced receptivity to mating [4,5]. Furthermore, females mated to males lacking SP show a diminished post-mating response, as they are more receptive to mating and lay fewer eggs than females mated to wild type males [6,7]. SP enters the female reproductive tract during copulation. A fraction of SP immediately circulates systemically by entering into the hemolymph [8], whereas another fraction remains bound to the tail of sperm and is thought to be gradually released into the hemolymph by cleavage [9,10]. SP binds the sex peptide receptor (SPR), a G protein-coupled receptor [11], which is expressed in the female reproductive organs and nervous system. SPR is required in a subset of sensory neurons in the reproductive tract to elicit the post-mating response [12,13]. Additionally, it has been shown that after mating females increase their food intake and have a preference for food with yeast, a source of proteins, which is thought to facilitate increased egg production. This change in food preference and intake requires SPR and receipt of SP [14-16].

Females that receive seminal fluid proteins, but no sperm, have a short-term post-mating response that lasts about a day, with increased egg production and decreased receptivity to mating. Long-term post-mating changes, which last about a week, require receipt of both sperm and seminal fluid proteins, with SP playing a critical role [9,10]. The slow release of SP that is bound to sperm tails is thought to elicit the long-term female post-mating changes. Other Acps contribute to the post-mating response in different ways including increasing ovulation rate (Acp26Aa, also called Ovulin; [17]) and sperm storage (Acp36DE; [18]). Additionally, *CG33943 *is required for full stimulation of egg laying, and four additional Acps (*CG1652, CG1656, CG17575, CG9997*) are required for sustained egg laying, release of sperm from storage organs, and reduced receptivity to courtship [10]. The functions of some 100 other Acps are largely unknown.

Despite having knowledge regarding the functions of seminal fluid proteins that contribute to the post-mating response, little is known about the downstream effector genes that mediate the post-mating response in females, especially in tissues outside of the reproductive tract, such as head and central nervous system (CNS) tissues. Microarray studies have determined the global changes in transcription observed after mating by comparing gene expression between virgin and mated females in whole animals [19-21] and in dissected female reproductive tract tissues [22]. It is clear that there are tissue-specific, post-mating gene expression responses [compare [19,22]; however, it is unlikely that changes in gene expression in head and brain tissues would have been detected by the previous whole animal studies given the sensitivity of the microarray assay [see [23,24]. Finally, while the post-mating response lasts for about a week, previous genomic studies only examined time points within 24 hours of copulation. Important, unanswered questions remain, including the identification of tissue-specific gene expression changes that occur in head and brain tissues post-mating and understanding how the post-mating response is maintained long-term.

Here, we present the results of a time-course study examining gene expression in head and dissected brain tissues of female flies after mating. Genes whose products function in metabolism show marked changes in expression levels in the head post-mating. A large proportion of these genes are expressed in the head fat body, a tissue that is similar to mammalian adipose tissue. The fat body can influence tissues at a distance through the release of factors into the hemolymph [25], as well as respond to energy needs by altering the storage of energy reserves, such as glycogens, lipids, and proteins [26]. We also identified genes that are expressed in the brain whose transcript abundance changes post-mating, including at least five genes that encode products with ion channel activity, suggesting that changes in neurophysiology mediate aspects of the post-mating response. Comparisons of the genes identified in this study with those previously identified in studies examining gene expression changes post-mating in whole animal or internal reproductive tissues revealed little overlap, highlighting that different tissues have a unique post-mating response at the level of gene expression.

## Results

We performed gene expression analyses to identify genes differentially expressed between virgin and mated females, in adult head and brain tissues. RNA was derived from age-matched, adult head tissues from virgin and mated females at 0-2, 24, 48, and 72 hours post-mating, and from brain tissues at 24 hours post-mating. For each time point, two-color, glass-slide microarray experiments were performed, using four independent biological replicates [for array platform [27]. All females were five days old when mated and raised under constant environmental conditions, and therefore, were expected to show minimal variation in gene expression levels [28]. Given this, most gene expression differences observed in these experiments are expected to be due to the post-mating response, rather than due to changes in gene expression in virgin female animals. We refer to genes with higher expression in mated females than in age-matched virgins as induced or up-regulated, and those with lower expression in mated females as repressed or down-regulated.

At the four time points examined, differences in the numbers of genes that showed significant differential expression between mated and virgin female head tissues were observed [0-2 (237 genes), 24 (326 genes), 48 (449 genes), and 72 (545 genes) hours post-mating, *P *< 0.05], with later times showing greater differences (Table 1). These results are distinct from those obtained from experiments that analyzed post-mating gene expression changes in whole adult females, in which thousands of genes showed significant expression differences early, at 1-3 hours post-mating, whereas only hundreds of genes were differentially expressed at each later time point, including 6, 12, and 24 hours after mating [20]. The post-mating changes in gene expression in head tissues were also distinct from those identified in the female reproductive tract tissues, in which the most differentially expressed genes were identified at 6 hours post-mating, with fewer identified at an earlier and later time point, 3 hours and 24 hours post-mating, respectively [22].

**Table 1:** The number of genes with significant differences in expression in head tissues post-mating at four time points (*P *< 0.05).

Time point	Number of genes
0-2 hours post-mating	
lower in mated females	93
higher in mated females	144
	
24 hours post-mating	
lower in mated females	208
higher in mated females	118
	
48 hours post-mating	
lower in mated females	283
higher in mated females	166
	
72 hours post-mating	
lower in mated females	316
higher in mated females	229

The difference in kinetics of the transcript changes likely reflects the diverse causes of gene expression changes in the tissues examined. Multiple factors might contribute to expression changes, including social and physical interactions [29], responses to individual seminal fluid components and sperm [20], and responses to fertilization and egg-laying [30]. Additionally, each tissue-type examined likely responds in a tissue- and temporal-specific manner. Given the experimental design, we are unable to distinguish between these different influences on gene expression changes.

### Biological processes that underlie the post-mating response in head tissues

**Figure 1:**
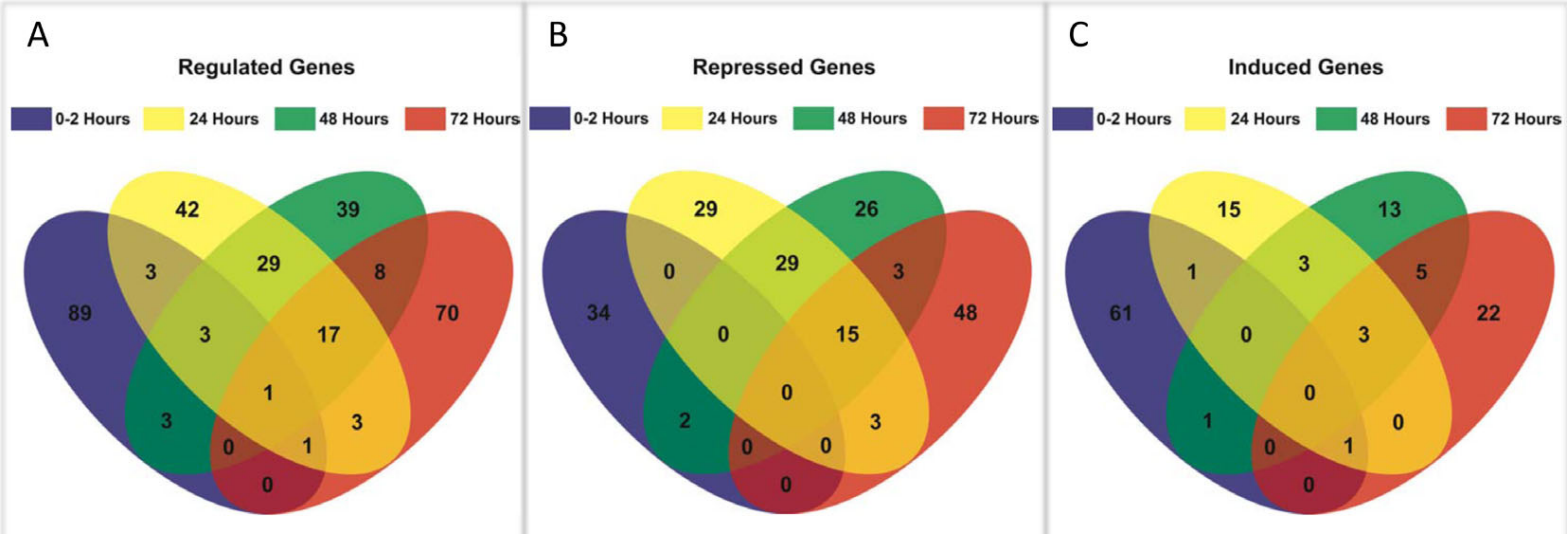
**Venn Diagrams showing the number of genes with significant differential expression that overlap among the four post-mating time points analyzed**. The top 100 ranked FDR genes identified as differentially expressed 0-2 (blue), 24 (yellow), 48 (green) or 72 (red) hours post-mating were used in the analyses. The number of all genes that overlap (A), only the genes that are down-regulated post-mating (B), and only the genes that are up-regulated post-mating (C) among the four time points is shown and indicated by the overlapping colors.

To gain insight into the types of biological processes that are responsive to mating in adult head tissues, we focused our analysis on the top 100 genes which showed significantly altered expression, based on false-discovery rank (FDR), from each time point (Additional file 1). Collectively, there are 309 unique genes from all four time points among the top 100 FDR ranked genes (genes present in multiple lists are counted once; Additional file 1). The genes identified at the 24 and 48 hour post-mating time points show the largest amount of overlap (50 genes overall; six up-regulated and 44 down-regulated; Figure 1). Not surprisingly, the genes from 0-2 and 72 hours stages show the least amount of overlap (two genes; one up-regulated and one that is significantly different at both time points, but regulated in opposite directions; Figure 1).

Only one gene, *CG8147*, is among the top 100 FDR ranked genes at all four stages; *CG8147 *encodes a product with predicted alkaline phosphatase activity [31]. *CG8147 *is significantly induced at all post-mating stages in head and brain tissues examined, except at the 0-2 hour stage in head tissues, where it is significantly repressed. In our previous study, we found that *CG8147 *shows significant female-biased expression in head tissues [24], perhaps due to up-regulated expression in females post-mating. Others studies show that *CG8147 *is highly repressed when flies are starved and suggest that there is a trade-off between nutrient status and reproductive status [32].

Examination of the Gene Ontology (GO) functional groups in the list of 309 genes allows for a global view of post-mating gene expression changes and reveals that changes in transcript abundance of genes that function in metabolism accompany the physiological changes that occur post-mating in head tissues (Table 2 and Additional file 1 identified using Flymine [33]). Nearly all of the 75 significant GO functional categories identified, including those most significantly overrepresented, contain genes that encode products involved in metabolic processes, including metabolism of amino acids, carbohydrates, and nucleotides (Table 2). We were not able to identify metabolic pathways for which all, or most, genes encoding enzymes in the pathway show expression differences in this study. This may be because each gene in the pathway is not regulated at the level of gene expression, or alternatively, because expression differences for these genes do not fall within the top 100 FDR ranked genes.

**Table 2:** The Gene Ontology functional enrichment of the genes identified as mating responsive in head tissues from all four time points

Most significant functional categories identified	
GO Functional Category	*P *value
small molecule metabolic process	2.02E-10
organic acid metabolic process	5.58E-10
carboxylic acid metabolic process	5.58E-10
oxoacid metabolic process	5.58E-10
cellular ketone metabolic process	3.16E-09
cellular amine metabolic process	4.44E-08
cellular amino acid metabolic process	1.39E-07
oxidation reduction	3.83E-07
cellular amino acid and derivative metabolic process	5.36E-07
alcohol metabolic process	1.16E-06
pentose-phosphate shunt	2.10E-05
NADP metabolic process	2.10E-05
NADPH regeneration	2.10E-05
amine biosynthetic process	3.29E-05
alcohol catabolic process	4.58E-05
amine metabolic process	5.95E-05
coenzyme metabolic process	1.12E-04
serine family amino acid metabolic process	1.14E-04
glycine metabolic process	1.18E-04
nicotinamide metabolic process	1.19E-04
alkaloid metabolic process	1.19E-04
nicotinamide nucleotide metabolic process	1.19E-04
cellular amino acid biosynthetic process	1.38E-04
pyridine nucleotide metabolic process	1.83E-04
small molecule catabolic process	2.01E-04

### Gene expression in head tissues 0-2 hours post-mating

Of the top 100 FDR ranked genes, 36 genes are repressed and 64 genes are induced 0-2 hours post-mating (Additional file 1). Of these 100 genes, eight repressed and 18 induced genes show at least two-fold differences in expression (Table 3).

**Table 3:** The five genes with the most substantial differences in expression that are induced and repressed post-mating at each time point.

Higher in females 0-2 hours post-mating				
Flybase	symbol	Fold-change	FDR	FDR rank
FBgn0052350	*CG32350*	2.67	1.54E-03	13
FBgn0004396	*CrebA*	2.77	7.49E-04	10
FBgn0086265	*Fcp26Aa*	4.83	1.31E-02	65
FBgn0035741	*BBS1*	5.44	1.72E-04	5
FBgn0033521	*CG12896*	2.56	1.15E-05	1
Lower in females 0-2 hours post-mating				
Flybase	symbol	Fold-change	FDR	FDR rank
FBgn0001225	*Hsp26*	11.98	1.50E-04	4
FBgn0035752	*CG8616*	10.91	3.19E-05	2
FBgn0001230	*Hsp68*	7.08	3.78E-05	3
FBgn0001228	*Hsp67Bb*	3.22	4.25E-04	6
FBgn0014018	*Rel*	2.55	1.57E-02	85
Higher in females 24 hours post-mating				
Flybase	symbol	Fold-change	FDR	FDR rank
FBgn0036381	*CG8745*	2.56	8.35E-03	80
FBgn0003250	*Rh4*	2.67	3.39E-03	42
FBgn0040074	*retinin*	3.13	5.14E-03	62
FBgn0040609	*CG3348*	3.56	3.00E-04	7
FBgn0043791	*CG8147*	4.39	8.76E-03	84
Lower in females 24 hours post-mating				
Flybase	symbol	Fold-change	FDR	FDR rank
FBgn0002565	*Lsp2*	18.47	4.53E-06	1
FBgn0002563	*Lsp1beta*	12.14	1.00E-04	2
FBgn0039330	*CG11909*	9.05	1.26E-04	3
FBgn0039685	*Obp99b*	5.44	1.31E-03	26
FBgn0040349	*CG3699*	5.32	8.31E-04	19
Higher in females 48 hours post-mating				
Flybase	symbol	Fold-change	FDR	FDR rank
FBgn0040502	*CG8343*	3.52	9.05E-06	10
FBgn0039703	*CG7829*	3.66	1.51E-05	15
FBgn0085353	*CG34324*	5.32	3.98E-05	24
FBgn0030773	*CG9676*	6.70	5.34E-07	4
FBgn0043791	*CG8147*	6.96	4.31E-04	47
Lower in females 48 hours post-mating				
Flybase	symbol	Fold-change	FDR	FDR rank
FBgn0002565	*Lsp2*	16.60	9.39E-08	1
FBgn0002563	*Lsp1beta*	9.10	3.69E-07	2
FBgn0039685	*Obp99b*	9.09	1.93E-05	17
FBgn0033830	*CG10814*	6.59	1.66E-05	16
FBgn0030073	*CG10962*	5.41	2.73E-05	20
Higher in females 72 hours post-mating				
Flybase	symbol	Fold-change	FDR	FDR rank
FBgn0037612	*CG8112*	2.17	4.10E-04	20
FBgn0052523	*CG32523*	2.35	7.54E-05	7
FBgn0043791	*CG8147*	3.07	2.01E-03	67
FBgn0000165	*Bc*	3.52	8.10E-05	8
FBgn0040609	*CG3348*	5.84	3.01E-06	2
Lower in females 72 hours post-mating				
Flybase	symbol	Fold-change	FDR	FDR rank
FBgn0033830	*CG10814*	7.87	2.67E-06	1
FBgn0039685	*Obp99b*	5.65	3.98E-06	3
FBgn0039298	*to*	4.18	1.01E-05	5
FBgn0031432	*Cyp309a1*	3.27	9.35E-05	10
FBgn0029158	*Las*	3.11	8.46E-05	9

The rapidity of the gene expression response suggests that Acps, or other small molecules contained in the seminal fluid, rapidly circulate in the female's hemolymph and influence gene expression in the head. The mode of signaling may be either direct, as SP has been found associated with brain tissues [34], or indirect, by influencing the physiology of other tissues, like the internal reproductive tissues and associated neurons, which may produce signaling molecules that influence gene expression at distant sites in the body [30]. Given how recently mating had occurred before collecting animals at this time point, the gene expression changes could also be due to social interactions, as it has been shown that males that have courtship interactions, but do not mate, have immediate gene expression changes [29].

### Genes with lower expression in females 0-2 hours after mating

Genes that are repressed 0-2 hours after mating include those that encode products involved in stress, immunity, or detoxification (*Heat shock protein 26, Heat shock protein 68, Heat shock gene 67Bb, relish, Glutathione S transferase E1*; Table 4 and Additional file 2 for biological enrichment). Expression of these genes might normally be high in virgin females, with part of the early post-mating response being a reduction of their expression. This result is in contrast to post-mating gene expression changes in whole animals where genes that encode products involved in immunity were up-regulated post-mating [20]. Additionally, SP induces the innate immune response [35]. One possible explanation for this discrepancy is that exposure to CO_2_, the anesthetic used here, induces a stress response. While our experimental design controlled for this by exposing both mated and unmated females to CO_2_, it is possible that a small increase in the exposure time of virgin females as compared to mated females could account for the apparent repression of genes with products involved in immunity. At all other time points examined flies had at least 24 hours to recover from CO_2 _exposure and work in our lab has demonstrated that CO_2 _exposure has no effect on gene expression after 8 hours (unpublished data).

**Table 4:** The ten most significant GO categories for genes with significant expression differences at each time point

Higher in mated females 0-2 hours post-mating		
GO Term	P value	Number of genes
dopamine metabolic process	4.72E-04	2
anatomical structure development	9.53E-04	18
developmental process	1.26E-03	20
catecholamine metabolic process	2.04E-03	2
catechol metabolic process	2.05E-03	2
phenol metabolic process	2.05E-03	2
diol metabolic process	2.05E-03	2
multicellular organismal process	2.29E-03	22
system development	3.08E-03	15
positive regulation of multicellular organism growth	3.69E-03	2
Lower in mated females 0-2 hours post-mating		
GO Term	P value	Number of genes
response to abiotic stimulus	2.18E-05	6
response to heat	9.81E-04	3
response to temperature stimulus	1.44E-03	3
alcohol metabolic process	1.66E-03	4
steroid catabolic process	3.17E-03	1
cholesterol catabolic process	3.18E-03	1
prenol metabolic process	3.19E-03	1
prenol biosynthetic process	3.20E-03	1
polyprenol metabolic process	3.20E-03	1
polyprenol biosynthetic process	3.21E-03	1
Higher in mated females 24 hours post-mating		
GO Term	P value	Number of genes
response to light stimulus	5.95E-04	3
response to radiation	9.19E-04	3
absorption of visible light	1.98E-03	1
arginine catabolic process to glutamate	1.98E-03	1
positive regulation of biosynthetic process	2.68E-03	3
positive regulation of cellular biosynthetic process	2.68E-03	3
visual perception	2.86E-03	2
sensory perception of light stimulus	3.01E-03	2
positive regulation of cellular metabolic process	3.33E-03	3
positive regulation of metabolic process	3.47E-03	3
Lower in mated females 24 hours post-mating		
GO Term	P value	Number of genes
small molecule metabolic process	3.61E-12	26
organic acid metabolic process	6.94E-11	16
carboxylic acid metabolic process	6.95E-11	16
oxoacid metabolic process	6.95E-11	16
cellular ketone metabolic process	2.16E-10	16
pentose-phosphate shunt	1.24E-07	4
NADP metabolic process	1.24E-07	4
NADPH regeneration	1.24E-07	4
nicotinamide metabolic process	7.35E-07	4
alkaloid metabolic process	7.37E-07	4
Higher in mated females 48 hours post-mating		
GO Term	P value	Number of genes
phosphatidylserine metabolic process	2.23E-03	1
phosphatidylserine biosynthetic process	2.24E-03	1
male courtship behavior	2.25E-03	1
arginine catabolic process to glutamate	2.25E-03	1
D-ribose metabolic process	4.55E-03	1
arginine catabolic process	4.57E-03	1
mating behavior, sex discrimination	4.57E-03	1
cellular process	9.92E-01	8
Lower in mated females 48 hours post-mating		
GO Term	P value	Number of genes
small molecule metabolic process	1.40E-12	26
organic acid metabolic process	4.52E-10	15
carboxylic acid metabolic process	4.54E-10	15
oxoacid metabolic process	4.54E-10	15
cellular ketone metabolic process	1.31E-09	15
hexose metabolic process	7.43E-09	9
monosaccharide metabolic process	2.86E-08	9
pentose-phosphate shunt	1.10E-07	4
NADP metabolic process	1.10E-07	4
NADPH regeneration	1.10E-07	4
Higher in mated females 72 hours post-mating		
GO Term	P value	Number of genes
phosphatidylserine metabolic process	3.14E-03	1
phosphatidylserine biosynthetic process	3.14E-03	1
leucyl-tRNA aminoacylation	6.34E-03	1
asparagine metabolic process	6.37E-03	1
asparagine biosynthetic process	6.37E-03	1
organic acid metabolic process	7.63E-03	4
carboxylic acid metabolic process	7.67E-03	4
oxoacid metabolic process	7.67E-03	4
biological regulation	9.97E-01	2
Lower in mated females 72 hours post-mating		
GO Term	P value	Number of genes
oxidation reduction	8.05E-06	13
organic acid metabolic process	3.75E-05	9
carboxylic acid metabolic process	3.76E-05	9
oxoacid metabolic process	3.76E-05	9
aspartate metabolic process	4.36E-05	2
cellular ketone metabolic process	6.82E-05	9
purine base biosynthetic process	2.61E-04	2
glutamate biosynthetic process	4.35E-04	2
small molecule metabolic process	5.26E-04	13
'de novo' IMP biosynthetic process	6.53E-04	2

### Genes with higher expression in females 0-2 hours after mating

Dopamine is required for female receptivity to courtship [36] and accordingly, genes that are up-regulated in response to mating include those involved in dopamine metabolism (Table 4 and Additional File 2). *pale*, which encodes a tyrosine hydroxylase important for the production of dopamine, was up-regulated immediately after mating (1.65 fold-change [FC]; FDR rank 25). This is consistent with dopamine playing a role in female receptivity, as the 0-2 hour time point includes females that were recently receptive to courtship. Since acetylation is thought to lead to dopamine inactivation, the up-regulation of *dopamine N-acetyltransferase *(1.43 FC; FDR rank 42), which converts dopamine to N-acetyl dopamine [37,38], provides a mechanism to achieve a subsequent reduction in dopamine activity.

*female-independent-of-transformer *(*fit*) showed increased expression in 0-2 hour post-mated head tissues (1.60 FC; FDR rank 15), whereas *fit *showed decreased expression at later times. Other studies examining the post-mating gene expression response in whole females also found significantly increased *fit *expression at several stages [19,21], consistent with *fit *playing a key, but unknown role in the post-mating response in many tissues. The biochemical function of Fit is not known, but *fit *is highly expressed in the fat body [39]. Furthermore, *fit *has also been shown to be down-regulated in response to starvation [32] and up-regulated in males in response to courtship interactions [29]. Taken together, *fit *appears to mediate or respond to reproductive and nutrient status in both males and females.

Additional genes that are up-regulated early in response to mating include *diminutive*, which encodes a transcriptional regulator important for animal growth and development (*dm*, also known as *dMyc*; 1.74 FC; FDR rank 52; [40]). Also, one *fruitless *isoform (microarray probe for the A DNA binding domain) is up-regulated in response to mating (2.04 FC; FDR rank 21). *fruitless *isoforms common to both sexes, including those with the A DNA binding domain, are expressed in the adult brain and may play a role in neural patterning [41,42]. In addition, the gene list includes *short neuropeptide F precursor *(2.02 FC; FDR rank 76), which encodes a neuropeptide that regulates food intake [43]; feeding is expected to increase as the females increase their egg-laying [44], and from our results this appears to be an early post-mating response.

### Gene expression in head tissues 24-hours post-mating

Of the top 100 FDR ranked genes, 77 genes are repressed and 23 genes are induced 24 hours post-mating (Table 4 and Additional file 2 for biological enrichment). Of these 100 genes, 59 repressed genes and 12 induced genes show at least two-fold differences in expression (Table 3 and Additional file 1).

The genes *larval serum protein 2 *(*Lsp2*) and *Lsp1beta *are two of the most highly repressed genes (Table 3), suggesting that a major shift in metabolic state is part of the post-mating response, as the products of these genes function as fat body nutrient reservoirs for the storage of amino acids [26,31]. In addition, *CG8147*, which is the gene that is the most highly induced in head tissues of mated females, also encodes a product involved in metabolic processes and has predicted alkaline phosphatase activity [31].

### Genes with lower expression in females 24 hours after mating

Analyses of the gene ontologies of the 77 repressed genes supports the reduced expression of metabolic genes post-mating. Nearly all of the 115 biological process categories significantly overrepresented are metabolic categories (Additional file 1). Furthermore, 19 genes were also identified in studies examining gene expression after 24 hours of starvation [identified using Flymine [33,45]]. These analyses support the idea that in head tissues a shift in metabolic state, nutrient utilization, and storage is involved with the post-mating response as early as 24 hours post-mating.

As validation of the experimental approach, 57 of the 77 genes repressed in head tissues at the 24-hour post-mating stage have been identified as highly expressed in the adult head (identified using Flymine [33]), by studies that examined tissue-specific gene expression [46]. Sixty of the 77 genes were identified as expressed in the adult fat body, suggesting that they are also likely expressed in the head fat body in this study. Many of the 77 genes are expressed in both mated and virgin spermatheca female tissues (51 and 60 genes, respectively). This latter observation can be reconciled by the fact that both the head and spermatheca tissues either contain or are closely associated with fat body tissue. Genes that function in the nervous system were also identified, including 12 genes expressed in the brain and 11 genes expressed in the ventral nerve cord.

### Genes with higher expression in females 24 hours after mating

Surprisingly, of the 23 genes with higher expression in mated females, 11 genes have enriched expression in the adult eye [46], or are known to function in visual transduction (include *rhodopsin 4, rhodopsin 5, retinin *and *trp-like*). This suggests that changes in the visual transduction system may accompany aspects of the post-mating response, which is an unexpected observation. It is also possible that the products of these genes have roles in other head tissues and that the change in their expression may not affect visual transduction, but rather, other physiological processes. Finally, the increased expression of these genes may reflect a change post-mating in the relative abundance of eye tissue, as compared to other head tissues, like the fat body. Our observation that there is a substantial repression of gene expression in the fat body post-mating may reflect that there is a decrease in fat body tissue in the head post-mating. Given the way microarray data are normalized, this could result in an apparent increase in expression of genes in other tissues, like the eye.

### Gene expression in head tissues 48-hours post-mating

Of the top 100 FDR ranked genes, 75 genes are repressed and 25 genes are induced 48 hours post-mating (Table 4 and Additional file 1 and 2). Of these 100 genes, 39 repressed and 14 induced genes show at least two-fold differences in expression (Table 3 and Additional file 1 and 2).

The genes *Lsp2 *and *Lsp1beta*, which encode nutrient reservoirs, are again among the most strongly repressed genes in head tissues of mated females (Table 3), as are *takeout (to; *2.86 FC; FDR rank 7), and *Odorant binding proteins 99a *(*Obp99a*; 4.02 FC; FDR rank 9) and *Obp99b *(9.09 FC; FDR rank 17; Table 3).

*to *expression is also repressed in adult head tissues in response to mating at 24 and 72 hours post-mating. This result is consistent with the previous finding that the *to *encoded product, which is highly expressed in the adult fat body, is more abundant in the hemolymph of males than in females [25]. *to *has previously been shown to function in the behavioral response to starvation, adult feeding behavior, male courtship behavior, and *to *expression is under circadian regulation [25,47-51]. The results presented here suggest another behavioral and physiological role for *to *in the female post-mating response.

In contrast to the results presented here, *Obp99a *was found to be more highly expressed in mated females at two time points (1-3 hours and 12 hours post-mating; [19]), when the whole animal was assayed, supporting the idea that different tissues have different gene expression responses post-mating. It has been shown in one study that *Obp99b *has substantial male-biased expression in head tissues [39], however in our previous study we observed no significant sex-biased expression in head tissues [24]. Also, *Obp99b *is up-regulated in males that have courted females [29], is higher in *fruitless *mutant males that display very reduced courtship behaviors (2.56 FC; FDR rank 4; [24]), and is down-regulated in males that have been starved [32]. In females, ectopic expression of *Obp99b *leads to reduced virgin female receptivity and copulation frequency [39]. Taken together, this suggests that expression of *Obp99b *is sensitive to and perhaps influences nutrient status and reproductive status in both males and females.

### Genes with lower expression in females 48 hours after mating

Of the 75 genes that are repressed 48 hours after mating, 44 were also repressed 24 hours after mating and include many of the genes involved in metabolism [identified using Flymine [33]]. The remaining 31 repressed genes at 48 hours, but not at 24 hours, are enriched with genes whose products are involved in glycogen and glucan metabolism and biosynthesis (*p *< 0.0001, two genes). This suggests that the timing of aspects of the metabolic shift observed post-mating depends on the biosynthetic pathway. Additional repressed genes in mated females at 48 but not at 24 hours include *Obp99a *and *Neuropeptide-like precursor 3 *(*Nplp3; *2.13 FC; FDR rank 49), which in contrast to this study, was induced post-mating when whole animal tissues were examined [19]

### Genes with higher expression in females 48 hours after mating

Of the 25 genes induced in mated head tissues at 48 hours, six were also induced at 24 hours and include genes encoding metabolic enzymes. The 19 remaining genes include seven genes that are expressed in the nervous system *(fruitless, Regulator of G-protein signaling 7, out at first, CG8745, Crk, CG11425*, and *CG11347)*, as assessed using Flyatlas data [46]. Overall, the gene expression changes observed at 24- and 48-hours post-mating are similar, however, more genes with known expression in the CNS are induced at 48 hours.

### Gene expression in head tissues 72-hours post-mating

Of the top 100 FDR ranked genes, 69 genes are repressed and 31 genes are induced 72 hours post-mating (Table 4 and Additional file 1 and 2). Of these 100 genes, 18 repressed and 8 induced genes show at least two-fold differences in expression (Table 3 and Additional file 1). The observation that there are fewer genes with robust differences in expression at 72 hours (FC >2), as compared to 24 and 48 hours, suggests that mating-induced transcriptional changes begin to diminish in magnitude after 48 hours.

The genes *Lsp2*, *to*, *Obp99a*, and *Obp99b *are again among the most repressed genes, as they were at 24 hours (Table 3). *CG3348 *is the most highly induced gene at 72 hours in mated head tissue (~ six-fold change) and was among the most highly induced genes at 24- and 48-hours post-mating. *CG3348 *encodes a product predicted to be involved in chitin metabolism [31], which suggests that changes in the regulation of cuticle synthesis underlie aspects of the female post-mating response, perhaps including the cuticle that forms the head capsule or the cuticle that is part of the adult trachea, the fly respiratory system. *CG3348 *was also shown to have greater than two-fold up-regulated expression in mated females, which is induced by sperm transfer in the absence of Acps [20].

### Genes with lower expression in females 72 hours after mating

Of the 69 genes that were repressed 72 hours post-mating, 19 were also repressed at 48 hours post-mating and include genes involved in metabolism. The remaining 50 genes that are repressed post-mating are enriched for those involved in immune response function (antibacterial humoral response; *p *< 0.0008; three genes; Additional file 1). Several genes involved in lipid metabolism are repressed at this stage (*brummer*, *doppelganger von brummer*, *secretory Phospholipase A2*). The observation that *Insulin-like peptide 5 *(1.51 FC; FDR rank 62) is in this gene set suggests that the metabolic state of head tissues is transmitted systemically to elicit responses in different tissues, as has been shown for *Insulin like peptides *produced in the head that influence egg production [52].

### Genes with higher expression in females 72 hours after mating

Of the 31 genes induced 72 hours post-mating, eight were also more abundant at 48 hours. The remaining 23 include *sugarbabe *(1.49 FC; FDR rank 42), a zinc-finger transcription factor that is induced in fat body and gut tissues in response to sugar [45], suggesting that sugar levels are higher in head tissues at 72-hours post-mating than at earlier times [45].

### Gene expression in dissected brain tissue 24-hours post-mating

To more specifically identify genes that play a role in the CNS to mediate post-mating behavioral changes, gene expression analyses were performed on dissected brain tissues 24 hours post-mating. Of the top 100 FDR ranked genes, 28 genes are repressed and 72 genes are induced in brain tissue 24 hours after mating. Of these 100 genes, five repressed and 20 induced genes show at least two-fold differences in expression (Table 2 and Additional file 1).

### Genes with lower expression in CNS tissues of females 24 hours after mating

Of the 28 genes that are repressed in dissected brain tissues 24 hours post-mating, only one gene, *Obp99b*, was also identified as repressed in whole head tissues 24 hours post-mating. This suggests that microarray experiments performed with RNA from whole head tissues lack the sensitivity to detect many gene expression changes in brain tissues. Twenty-two of the 28 genes have previously been shown to be expressed in brain tissues [46], validating the experimental approach. *shaker*, a voltage-gated cation channel, is among the genes that show the most substantial repression post-mating (2 FC; FDR rank 45). Five of the 28 genes have ion channel activity (*pHCl, shaker, cacophony, Ca*^*2+*^*-channel-protein-beta-subunit, tipE homolog 1*), suggesting that regulation of these genes is involved in the post-mating behavioral response. In addition, four of the 28 genes encode products with mRNA binding activity (*bruno-3, hephaestus, orb2, RNA-binding protein 6*), two encode products with axon guidance activity (*smooth, Protein tyrosine phosphatase 99A*) and one encodes a product with neurotransmitter transport activity (*complexin*) [31]. A set of critical neurons that respond to SP are not in the brain [12,13], but the gene classes identified here suggest that there are substantial changes in neurophysiology in the brain that may underlie post-mating behavioral changes.

### Genes with higher expression in CNS tissues of females 24 hours after mating

Of the 72 genes that are up-regulated in dissected brain tissues 24 hours post-mating, five genes were also identified as induced in whole head tissues 24 hours after mating; these include *retinin*, which is expressed in the visual system, and four additional genes with various functions (chitin binding, alkaline phosphatase activity and two of unknown function). Additionally, several genes known to be expressed in brain tissues were identified, including those encoding proteins that function in chromatin assembly (*dre4*), mRNA localization (*larsen*) and wingless protein binding (*nimrod B4*), suggesting that several different molecular mechanisms underlie the post-mating response in the brain. Four genes in this set are also involved in immune function. SP is known to induce the innate immune response [35,53], suggesting that SP or other factors can elicit an immune response in the brain.

### Expression of mating-responsive genes in different tissues

To determine how similar the post-mating response is in different tissues, we compared the 309 genes identified here as having post-mating gene expression changes in head tissues with genes identified in previous studies that examined different tissues or whole animals. Only four genes identified here (*fit*, *CG6910, Lsp1beta*, *psd) *were also identified as differentially expressed in mated versus virgin whole females [19]. Adult reproductive tissues may express a similar group of mating-responsive genes as head tissues, given that many genes identified here as expressed in head tissues are also expressed in spermathecae. Furthermore, eighteen genes identified here were previously identified as differentially expressed in mated and virgin female reproductive tissues (Additional file 3) [22]. In both comparisons noted, whether the gene is induced or repressed varies with the tissue and time point examined.

To determine whether the genes identified here as mating-responsive in female head tissues are also sex-differentially expressed, we compared genes identified in this study with our previous study that identified genes with sex-differential expression in head tissues [24]. Sixteen mating-responsive genes in females were also identified as having significant sex-differential expression. The sex-biased expression does not always conform to what might be expected; for example, a gene that is up-regulated post-mating might be expected to have female-biased expression, but only about half the genes show this pattern (Additional file 3). Therefore, most genes that are induced post-mating at the four times examined here do not show sex-differential expression in adults that are 0-24 hours old, which may also be due to differences in reproductive status and age between the two studies.

## Discussion

We have identified hundreds of genes that change expression post-mating in female head and brain tissues. At the four time points examined, a wide range of genes showed significant differential expression between mated and virgin female head tissues [0-2 (237 genes), 24 (326 genes), 48 (449 genes), and 72 (545 genes) hours post-mating, *P *< 0.05]. The largest functional group of significantly differentially expressed genes encodes products involved in metabolism that are expressed in the fat body. We observe that many of these genes are repressed starting at 24 hours post-mating, but not at the earlier 0-2 hour post-mating time point. This is likely due to the fact that egg production is energetically costly and females shift from nutrient storage to utilization, as their stores are depleted. For example, *Lsp1beta *and *Lsp2*, which encode amino acid reservoirs [26,31], are among the most highly repressed genes at 24 and 48 hours post-mating, but not at the 0-2 hour post-mating stage. Given that many of the changes in expression affect genes that encode products involved in metabolism, it is not clear if this is a direct response to receipt of sperm and seminal fluid, or due to social interactions that occur during courtship and mating. It is likely that for many genes the expression changes are secondary responses to the dramatic changes in physiology that occur due to increased egg laying. Additional experiments that assay gene expression changes in females that mate with males that lack sperm or accessory gland proteins will help distinguish between these possibilities, as will assaying gene expression in females that do not produce eggs, and in females that interact with males, but do not copulate.

Furthermore, it is not clear how many of the changes in gene expression in the head direct the female post-mating response, as opposed to being a consequence of changes in reproductive status; this is especially the case for genes with expression in head fat body. Other work has suggested that the head fat body may have additional roles beyond energetic support and might have complex interactions with the nervous system, ultimately influencing sex-specific behaviors. For example, a masculinized fat body is required for wild type levels of male courtship behavior [25]. Additionally, our previous work showed that *fruitless *mutations affecting male courtship behaviors also affect male gene expression in the head fat body [24]. Given that *fruitless *is expressed in the nervous system, this suggests that the physiology of the nervous system and/or the consequence of performing courtship behaviors influences gene expression in the fat body, which is supported by an additional study [29]. The idea that there are interactions between the fat body and the nervous system is also supported by the observation that silencing or activating the *fruitless*-expressing neurons that underlie sex-specific behaviors in both males and females can substantially shift fat content in adult flies [54]. Additionally, it has been shown that gene expression in the fat body affects female reproductive behaviors, with ectopic expression of *Obp99b *in female fat body tissues leading to reduced receptivity and mating success [39]. Taken together, one hypothesis is that there is a dynamic interplay between head fat body metabolic state and endocrine function and nervous system function that ultimately influence and direct adult reproductive behavior and physiology.

Gene expression changes in the brain are likely to mediate some of the long-term behavioral changes that occur post-mating, which lasts about one week and includes reduced receptivity to mating and increased egg laying. While much progress has been made in understanding at a molecular-genetic level how SP, SPR and other Acps influence the post-mating behavioral response [reviewed in [1,2]], most of the downstream effectors in the brain are not known, especially those that mediate the long-term behavioral female post-mating response. In this study, several genes with expression in the brain that encode products that function in neurophysiology were identified, with five having ion channel activities with known roles in behaviors [31]. An important next step will be to determine where these channels are expressed in females and how changes in their expression levels influence neurophysiology and behavior. To identify additional genes that mediate the post-mating response in brain tissue, it will also be important to assay gene expression in the nervous system at more time points, using more sensitive techniques, such as deep-sequencing technologies.

Many of the genes identified in this study with significant differential expression in head tissues were also identified as significantly enriched in gene lists from other expression studies (Benjamini and Hochberg *P *< 0.01; [assessed using Flymine [33]]), including those assaying circadian regulation of gene expression (22 genes [50]; 31 genes [55]), gene expression in response to nutrient status (15 genes [32], 17 genes [45]), sex-differential gene expression (9 genes [24]), and post-mating gene expression in whole animals (13 genes [19]). This overlap demonstrates that in the future it will be important to use an integrated, systems-level approach to understand this complex behavior.

## Conclusions

Substantial changes occur in the expression of genes in female head tissues after mating. These results provide new insights into the physiological and metabolic changes that accompany changes in female behaviors. By identifying the gene expression changes prompted by mating, this dataset provides a basis for future mechanistic studies that examine how specific genes mediate behavioral and physiological changes in Drosophila females post-mating. Additionally, understanding how these changes in gene expression orchestrate the post-mating response in Drosophila may provide insight into the reproductive behavior of more complex animals.

## Methods

### Drosophila stocks and husbandry

Flies were raised at 25°C under a 12-hour light and 12-hour dark cycle, on standard cornmeal food media. In all experiments, female flies were the wild-type Canton S strain. The male flies were the genotype *w; P[UAS-dj-GFP]*, which contains a transgene that expresses the Don Juan protein tagged with GFP (DJ-GFP) [56]. DJ-GFP associates with the sperm tail allowing visual confirmation of mating by the presence of GFP in the reproductive tract of mated females.

### Mating conditions and fly collections

Virgin Canton S females and *w; P[UAS-dj-GFP] *males were collected within five to eight hours of eclosion and aged for five days. On the fifth day, females and males were combined (ten females with 20 males) for two hours between the time period of 6-8 hours after the lights came on in the incubator. Females were then sorted under CO_2 _anesthesia using a fluorescence microscope. Only females with strong GFP expression in the lower abdomen, indicating the presence of sperm in the reproductive tract, were collected and aged on food. The mated females were then collected 24, 48 or 72 hours post-mating by tapping them without anesthesia into a cryovial, and immediately snap-freezing the flies in liquid nitrogen. Age matched virgin females were collected in a similar manner. For the 0-2 hour time point, animals were anaesthetized after the two hour mating period, identified as mated females and were immediately snap frozen in liquid nitrogen. The virgin females collected for the 0-2 hour time point were not exposed to males, but were anaesthetized to control for gene expression differences due to CO_2 _exposure, and then snap frozen. For experiments analyzing gene expression in dissected brain tissues, flies were mated as above and 24 hours after mating were lightly anaesthetized individually on ice. Brain tissue was dissected quickly in ice-cold 1× phosphate buffered saline (PBS) made with RNase-free water. The brain tissue was rinsed in 1× PBS to remove non-brain tissue and immediately placed in ice-cold Trizol (Invitrogen) until RNA extraction.

### RNA extraction and microarray experiments

The two-color, glass-slide microarray platform, labeled probe preparation and hybridization conditions were performed as previously described [27]. Adult heads were snapped-off from the body by shaking the frozen flies in the cryovial. The frozen heads were sorted from the bodies on plastic cooled on dry ice. Total RNA was extracted from ~100 heads per sample, using Trizol (Invitrogen). All experiments were performed with four independent biological samples, and a dye swap design to control for biases in dye incorporation. For experiments using RNA derived from head tissue, 20 μg of total RNA was used to generate labeled cDNA. For experiments using dissected brain tissue, 20 brains were dissected per sample and cRNA probes were made by converting the RNA to double stranded cDNA that is flanked by a T7 promoter. T7 RNA polymerase was then used to generate cRNA, using the Amino Allyl MessageAmp II aRNA Amplification Kit (Ambion).

### Microarray Data Statistical Analyses

Statistical analyses were performed as previously described [27]. To identify genes with significant differences in gene expression, a False Discovery Rate (FDR) method was employed. First *P *values were determined using the LIMMA package of BioConductor in the program R and converted to FDR values [57-59]. A hypergeometric statistical test was used to determine the significance of enrichment of functional groups, with the Benjamini and Hochberg multiple hypothesis test correction at a *p *< 0.01 threshold, implemented using Flymine [33]. For comparisons with the McGraw *et al. *2008 study, analyses were limited to genes from that study that showed > two-fold difference [19]. For comparisons with the Goldman and Arbeitman study, the analyses were limited to the top 100 FDR ranked genes with sex-differential expression in the same Canton S strain used here [24]. All data can be downloaded from the GEO website: GSE22390.

### Ethics statement

The research performed in this study on the fruit fly, *Drosophila melanogaster*, did not need to be approved by an ethics committee.

## Abbreviations

(SP): Sex peptide; (Acps): Accessory Gland Proteins; (Lsp): Larval serum protein; (CNS): Central Nervous System; (Obp): Odorant Binding Protein; (FDR): False Discovery Rate; (GO): Gene Ontology; (*fit*): *female-independent-of-transformer*; *(to)*: *takeout*; (GO): Gene Ontology.

## Authors' contributions

All of the authors contributed either to the data generation, data analysis, reagents, or paper writing. AN and ES performed the microarray experiments. ML performed the statistical analyses. SK generated the Venn Diagrams. All authors contributed to the analyses and writing and approved the final manuscript.

## Supplementary Material

Additional file 1 **FDR 100 and GO 309 genes**. The top FDR ranked genes with gene expression changes at 0-2, 24, 48 and 72 hours post-mating. The top FDR ranked genes with gene expression changes in brain tissues 24 hours post-mating. Gene Ontology functional enrichment of 309 genes that are the intersection of genes identified in head tissues at four time points assayed. Click here for file

Additional file 2 **GO for each list**. GO analyses of lists of top 100 FDR ranked genes from Additional file 1. Click here for file

Additional file 3 **Comparisons to other datasets**. Analysis of overlap between this study and Mack et al study and analyses of overlap between this study and Goldman et al study. Click here for file
